# Exploring the associations between data-driven insomnia disorder combined with mild anxiety or/and depressive symptoms and the efficacy of Cognitive-Behavioral Therapy for insomnia

**DOI:** 10.1016/j.ijchp.2025.100562

**Published:** 2025-04-08

**Authors:** Dongbin Lyu, Ruiyi Qian, Fangmei Ge, Yang Wang, Hongyan Wang, Yating Zhao, Hui Han, Ruyun Liu, Yutong Liu, Yiling Chen, Caojun Ji, Xin Luo, Tianhong Zhang, Yue Leng, Jie Zhang, Chengmei Yuan, Zeping Xiao

**Affiliations:** a Shanghai Mental Health Center, Shanghai Jiaotong University School of Medicine, Shanghai, China; b Department of Psychiatry and Behavioral Sciences, University of California, San Francisco, CA, USA

**Keywords:** Insomnia disorder, Cognitive-behavioral therapy, Cluster analysis, Subtypes, Efficacy

## Abstract

**Objectives:**

The endophenotype of insomnia disorder is complex and the treatment is not targeted. The data-driven typing method might provide some bases for precise treatment. The present study was based on a post hoc analysis, aiming to explore the association between subtypes of insomnia disorder and the efficacy of cognitive-behavioral therapy for insomnia (CBT-I).

**Methods:**

The present study was conducted on data of 118 patients with chronic insomnia disorder combined mild anxiety or/and depressive symptoms, who had completed an 8-week randomized controlled trial of CBT-I vs CBT-I plus (CBT-I combined with modules targeting anxiety and depressive symptoms). The silhouette coefficient determined the optimal number of clusters, and a K-means clustering analysis was performed. T-tests were conducted to assess baseline differences at eight weeks, and the changes in self-reported total sleep time (sTST), Pittsburgh Sleep Quality Index (PSQI), Hamilton Depression Rating Scale (HRSD-17), and Hamilton Anxiety Rating Scale (HAMA) scores in order to explore the impact of subtypes and treatment approaches (CBT-I and CBT-I plus) on insomnia and emotional symptoms.

**Results:**

The analysis revealed no significant demographic differences between the two clusters. Subtype 2 was characterized by significantly poorer baseline sleep quality (PSQI: 16.59 vs 12.74, *t* = -9.90, *p* < 0.01), higher depressive (HRSD: 18.47 vs 13.21, *t* = -8.37, *p* < 0.01), and anxiety levels (HAMA: 17.47 vs 13.46, *t* = -6.23, *p* < 0.01), and shorter sTST (4.67 vs 6.09 h, *t* = 8.31, *p* < 0.01) compared to Subtype 1. Post-treatment analyses showed significant improvements in both subtypes, with Subtype 2 experiencing a larger increase in sleep duration (csTST: 0.58 vs 1.77 h, *t* = -7.18, *p* < 0.01) and more pronounced improvements in sleep quality (cPSQI: 6.92 vs 8.88, *t* = -3.57, *p* < 0.001), depression (cHRSD: 8.07 vs 10.59, *t* = -2.71, *p* = 0.008), and anxiety (cHAMA: 9.28 vs 11.22, *t* = -2.56, *p* = 0.012). Despite these improvements, Subtype 1 maintained significantly better outcomes in sleep quality (PSQI: 5.81 vs 7.71, *p* < 0.01), depression (HRSD: 5.14 vs 7.89, *p* < 0.01), and anxiety (HAMA: 4.18 vs 6.25, *p* < 0.01) at 8 weeks. No significant differences in baseline characteristics were found between treatment groups within subtypes, indicating homogeneity. Within Cluster 1, CBT-I plus was more effective in reducing depressive symptoms (cHRSD: *t* = -2.48, *p* = 0.016), whereas CBT-I was superior in enhancing sTST in Cluster 2 (*t* = 2.01, *p* = 0.049), with no significant differences in other measures.

**Conclusions:**

The study underscores the heterogeneity within ID subtypes and the differential response of sleep quality and depressive symptoms to CBT-I and CBT-I plus, highlighting the importance of personalized treatment strategies based on insomnia subtypes.

## Statement of significance

Insomnia disorder is a heterogeneous disease. It is still controversial whether its treatment response varies by insomnia subtypes. The present study reveals a significant impact of data-driven insomnia subtypes on the efficacy of cognitive-behavioral therapy for insomnia (CBT-I). Different subtypes show varying improvements in insomnia and depressive symptoms by CBT-I or CBT-I combined with modules targeting anxiety and depressive symptoms. The findings of the present study reinforce personalized treatment approaches and highlight the importance of optimizing CBT-I interventions based on insomnia subtypes for improved outcomes.

## Introduction

Insomnia disorder (ID) is a prevalent sleep disturbance that is frequently comorbid with depression and anxiety, posing significant clinical challenges. A meta-analysis of 34 cohort studies, encompassing 172,077 participants, revealed a significant association between insomnia and an elevated risk of developing depression and anxiety. (Baglioni et al., 2011) This association was further corroborated by several subsequent studies (Chen et al., 2017; Hertenstein et al., 2019; Sivertsen et al., 2012), emphasizing the importance of addressing concurrent anxiety and depression symptoms in individuals with ID.

Furthermore, ID exhibits considerable heterogeneity with distinct symptom profiles (Benjamins et al., 2017). During the last few years, several studies have proposed various data-driven subtypes of insomnia (Crawford et al., 2017; Miller et al., 2016; Blanken et al., 2019; Hirakawa, 2019; Foley et al., 2010). For instance, a cluster analysis by Van De Laar et al. identified a phenotypes among 218 patients with insomnia, characterized by significant anxiety and depressive symptoms (Miller et al., 2016). These findings underscore the need for tailored treatment approaches that consider how diverse non-sleep symptoms influence insomnia.

Exploring the integration of additional interventions with cognitive behavioral therapy for insomnia (CBT-I) holds promise for addressing insomnia subtypes with depression and anxiety. CBT-I, whether delivered in-person or digitally, is the first-line treatment for insomnia in adults of all ages, including those with comorbidities (Riemann et al., 2023). While CBT-I has shown efficacy in treating both insomniac and emotional symptoms/psychiatric distress (Lau et al., 2022; Green et al., 2014), its effectiveness may be limited by the absence of specific interventions for comorbid depression or anxiety (Kraepelien et al., 2022). Classic CBT components targeting depression and anxiety, such as monitoring and addressing negative automatic thoughts (Gautam et al., 2020), could enhance the treatment of emotional symptoms when incorporated into CBT-I.

Previous studies exploring the impact of insomnia subtypes on treatment outcomes have certain limitations. It is concerning that nearly 40 % of patients do not achieve remission after cognitive-behavioral therapy for insomnia (CBT-I) treatment (Morin et al., 2020), which may be partly attributed to the lack of consideration for insomnia subtypes. However, studies using sleep indices (i.e., short or long sleep duration) to predict CBT-I effectiveness for ID have yielded inconclusive results (Bathgate et al., 2016; Lovato et al., 2016; Galbiati et al., 2020). Moreover, data-driven approaches have identified robust subtypes of insomnia disorder that were distinguished by biologically based traits and life history, rather than just sleep-related characteristics (Blanken et al., 2019). These subtypes have shown clinical relevance, including differing responses to treatment and varying risks of developing depression. Therefore, using data-driven subtypes to delineate non-sleep characteristics of ID, such as depressive and anxiety symptoms (Van Someren, 2021), may provide a more personalized and effective approach to treatment.

Therefore, the study aims to investigate the impact of data-driven subtypes of ID on the effectiveness of CBT-I. We hypothesize that the distinct depression, anxiety, and sleep profiles observed in the insomnia subtypes would respond differently to CBT-I, and that the effectiveness of both CBT-I and its augmented version, CBT-I plus, would vary by ID subtype. Ultimately, this investigation has the potential to augment the overall effectiveness of CBT-I and enhance the well-being of individuals with ID and its comorbidities.

## Methods

### Study design and participants

This study undertook analyses utilizing the data from a randomized controlled, evaluator-blind trial (NCT04585282) titled "CBT for Insomnia with Anxiety and Depression." The original research aims to investigate the effectiveness of an enhanced cognitive behavioral therapy for insomnia (CBT-I plus) compared to CBT-I in insomniac patients with symptoms of anxiety or depression. The participants were recruited from the sleep disorder clinic of Shanghai Mental Health Center in China from August 1, 2020, to March 1, 2023. The study design was approved by the ethical committee of Shanghai Mental Health Center. All participants signed an informed consent.

### Inclusion criteria

Inclusion criteria encompassed individuals between 18 and 65 years who, as evaluated by a research psychiatrist, met the diagnostic criteria for insomnia disorder according to DSM-5. Eligible participants had a total score of ≥ 10 on the Pittsburgh Sleep Quality Index (PSQI) and exhibited moderate levels of anxiety or depressive symptoms, with scores ranging from 14 to 29 on the Hamilton Anxiety Scale (HAMA) and/or scores ranging from 14 to 23 on the 17-item Hamilton Depression Scale (HRSD-17). To ensure consistency, participants refrained from using sedative hypnotics, antidepressants, anxiolytics, antipsychotics, or relevant medications for at least two weeks prior to enrollment, or they maintained stable medication type and dosage for at least four weeks before enrollment.

### Exclusion criteria

Those with other sleep disorders as specified by DSM-5 or other severe physical illnesses were excluded, as well as patients with substance abuse, pregnancy, a history diagnosis of depression, anxiety, or other mental disorders, or previous psychological therapy for a period exceeding three months.

### Interventions

Following informed consent, participants were randomly allocated in a 2:1 ratio to either the CBT-I plus intervention group (experimental group) or the CBT-I intervention group (control group). Ten uniformly trained therapists were randomly assigned to conduct the interventions. The therapy commenced with a comprehensive assessment, aimed at establishing therapeutic rapport, followed by eight weekly individualized sessions lasting 45–50 min each. The experimental group received the CBT-I plus program, while the control group received the standard CBT-I program. Table 1 provides an overview of the interventions, with detailed descriptions available in the Supplemental Methods. Throughout the intervention period, participants were required to maintain a stable dosage of their pre-existing medication and refrain from utilizing antidepressants, anxiolytics, or antipsychotic medications. Limited utilization of zopiclone, zolpidem (Ambien), or eszopiclone (Lunesta) was permissible, with a frequency not exceeding three times per week and a cumulative duration not surpassing two weeks.

**Table 1:** Comparison of Components in the Standard CBT-I and CBT-I plus Programs.

Session	Intervention	
	CBT-I	CBT-I plus
1	• Build rapport	• Build rapport
	• Introduce CBT-I	• Introduce CBT-I Plus
	• Sleep hygiene education	• Sleep hygiene education
	• Teach relaxation	• Teach relaxation
2	• Understand sleep mechanisms	• Understand sleep mechanisms
	• Analyze insomnia causes	• Analyze insomnia causes
	• Learn sleep restriction	• Learn sleep restriction
	• Develop sleep plan	• Develop sleep plan
3	• Evaluate benefits & adherence	• Evaluate benefits & adherence
	• Adjust sleep plan	• Adjust sleep plan
	• Promote behavior change	• Promote behavior change
4	• Adjust sleep plan	• Adjust sleep plan
	• Introduce stimulus control	• Introduce stimulus control
	• Mid-treatment summary	
5	• Continue sleep plan adjustment	• Continue sleep plan adjustment
	• Address adherence barriers	• Identify dysfunctional sleep beliefs
		• Correct anxiety-related thoughts
6	• Adjust sleep plan	• Adjust sleep plan
	• Correct sleep cognitions	• Challenge sleep beliefs
		• Correct depression-related thoughts
7	• Adjust sleep plan	• Adjust sleep plan
	• Continue cognitive restructuring	• Continue cognitive restructuring
	• Prepare for termination	• Prepare for termination
8	• Evaluate benefits	• Evaluate benefits
	• Discuss medication	• Relapse prevention
	• Relapse prevention	• End treatment
	• End treatment	

### Measures

We collected baseline demographic information, including age, gender, years of education, marital status, and body mass index (BMI). To assess sleep-related characteristics, we administered the Pittsburgh Sleep Quality Index (PSQI), which provided data on self-reported sleep quality and self-reported total sleep time (sTST), and other sleep indices (Sleep Latency; Sleep Efficiency; and Time in Bed). The PSQI ranges from 0 to 21, with higher scores indicating poorer sleep quality. Our primary analyses mainly focused on sTST to evaluate participants’ sleep quality. The severity of depressive and anxiety symptoms was quantified using the 17-item Hamilton Depression Rating Scale (HRSD-17) and Hamilton Anxiety Rating Scale (HAMA), respectively. The HRSD-17 ranges from 0 to 52, with higher scores reflecting greater severity of depressive symptoms, while the HAMA ranges from 0 to 56, with higher scores indicating more severe anxiety symptoms. Daytime sleepiness was measured with the Epworth Sleepiness Scale (ESS), which ranges from 0 to 24, with higher scores representing greater daytime sleepiness. We also used the Dysfunctional Beliefs and Attitudes about Sleep Scale (DBAS-16) to probe maladaptive cognitive patterns and attitudes about sleep; this scale ranges from 16 to 90, with higher scores suggesting more dysfunctional beliefs about sleep, which is contrary to the scoring rules used in most other studies. We conducted the assessments at baseline, including the PSQI, the HRSD-17, the HAMA, the ESS, and the DBAS-16. The PSQI, the HRSD-17. To evaluate CBT-I treatment outcomes, the HAMA were re-assessed at week 8.

### Data analysis

We calculated descriptive statistics to overview the participants' baseline demographic and clinical characteristics. We presented continuous variables as means and standard deviations, and express categorical variables as frequencies and percentages. For missing data, we employed multiple imputation. Specifically, the missing data proportions for the key variables were as follows: PSQI (0.85 %), HRSD (0.85 %), HAMA (0 %), DBAS (9.32 %), and ESS (9.32 %). Because some participants completed treatment but only partially filled out the self-reported scales, multiple imputation was chosen to retain as many cases as possible, minimize bias from missingness, and preserve statistical power—essential for subtyping analyses requiring full variable inclusion.

A data-driven methodology was implemented to discern subtypes within the study cohort. K-means clustering analysis was applied to baseline features, incorporating sTST and total scores of the PSQI, HRSD-17, HAMA, DBAS-16, and ESS. Before analysis, we standardized the features using z-score scaling to ensure comparability across different measurement scales. The optimal number of clusters, we selected the value that yielded the highest silhouette coefficient, which assesses the separation distance between resulting clusters. Subsequently, dimensionality reduction was performed using Principal Component Analysis (PCA), retaining components that accounted for at least 70 % of the variance in the data.

We used T-tests to compare baseline and follow-up characteristics between subtypes and among participants receiving different interventions. We assessed the changes in sTST (csTST), PSQI (cPSQI), HRSD (cHRSD), and HAMA (cHAMA) scores following the treatment. Specifically, csTST was calculated as the difference between the post-intervention and baseline values (post-intervention minus baseline), with positive values indicating an increase in total sleep time. In contrast, cPSQI, cHRSD, and cHAMA were calculated as the difference between the baseline and post-intervention values (baseline minus post-intervention), where positive values represent improvements, such as better sleep quality, reduced depressive symptoms, or reduced anxiety symptoms.

The analyses were conducted using Python 3.9 with packages "*sklearn*", "*scypi*", "*statsmodels*", "*pandas*", and "*numpy*". The visualizations were crafted using Python packages "*matplotlib*" and "Axes3D". We set α = 0.05 as the significance level to determine the statistical significance of the results.

## Results

### Baseline characteristics of the sample

Four hundred and eleven individuals were screened for the study. Among them, 76 individuals declined further evaluation, 187 individuals did not meet the inclusion criteria, and 148 eligible participants were included for further assessment and treatment. Ultimately, 118 individuals completed the 8-week intervention. The baseline demographic and clinical characteristics are shown in Table 2. Among the participants, 78 out of 118 participants (66.1 %) received CBT-I plus therapy. There were no significant differences in baseline characteristics between the CBT-I group and the CBT-I plus group.

**Table 2:** Baseline demographic and clinical characteristics.

	Intervention			
	CBT-I plus	CBT-I	t or *χ*²	P value
Age (years)*	35.18 (9.96)	34.92 (10.13)	−0.13	0.90
Education (years) *	16.46 (2.28)	16.89 (2.25)	0.95	0.35
Female#	46 (63.1 %)	23 (62.2 %)	0.00	1.00
Married#	33 (45.21 %)	17 (45.95 %)	0.14	0.93
BMI (kg/㎡) *	21.40 (3.05)	21.46 (2.77)	0.11	0.96
PSQI*	14.38 (2.93)	14.32 (2.76)	−0.10	0.92
Sleep Disturbance (PSQI) *	1.47 (0.55)	1.41 (0.61)	−0.45	0.66
sTST (mins) *	324.45 (69.35)	333.24 (76.09)	0.59	0.56
SL (min) *	65.73 (49.61)	69.00 (40.34)	0.37	0.71
SE (%)*	62.33 (14.92)	64.37 (14.40)	0.68	0.50
TIB (min) *	529.60 (110.01)	527.14 (70.26)	−0.14	0.89
HRSD-17*	16.32 (4.13)	14.57 (4.25)	−2.07	0.04
HAMA*	15.26 (3.90)	14.95 (4.40)	−0.37	0.72
DBAS-16*	35.81 (9.13)	32.91 (12.47)	−1.26	0.21
ESS*	6.66 (4.81)	5.57 (3.83)	1.20	0.23

Abbreviations: CBT-I, Cognitive Behavioral Therapy for Insomnia; PSQI, Pittsburgh Sleep Quality Index; sTST, Self-reported Total Sleep Time; SL, Sleep Latency; SE, Sleep Efficiency; TIB, Time in Bed; HRSD-17, 17-item Hamilton Rating Scale for Depression; HAMA, Hamilton Anxiety Rating Scale; DBAS-16, Dysfunctional Beliefs and Attitudes about Sleep; ESS, Epworth Sleepiness Scale.

⁎ : Continuous variables are presented as mean (standard deviation) and were analyzed using *t*-tests.

# : Categorical variables are expressed as n (%) and were analyzed using the χ² (chi-square) test.

### The data-driven clustering of the sample with ID

**Fig. 1:**
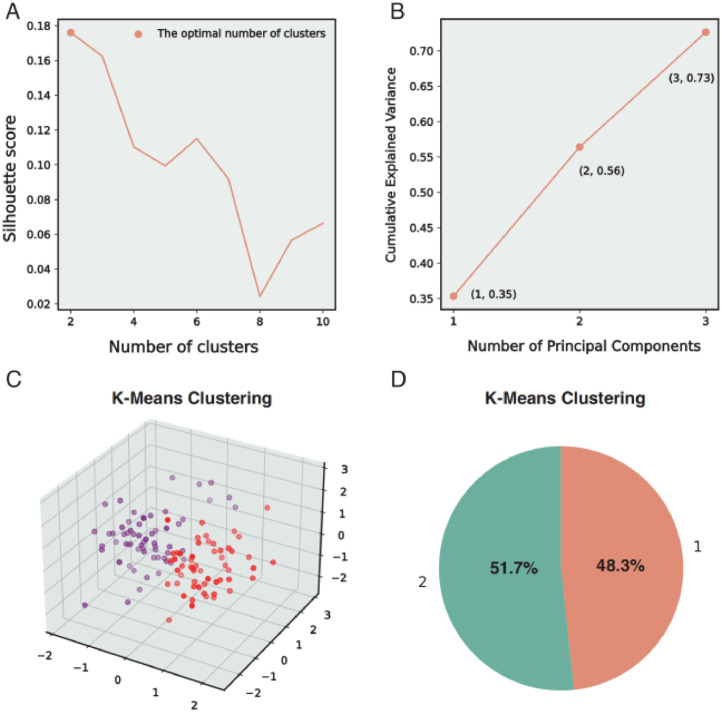
The visualization of the data-driven clustering process. (A) The silhouette coefficient score by number of clusters. (B) The principal component analysis (PCA) and the variance explained by the number of components. (C) The spatial representation of clusters formed through K-means clustering in three dimensions. (D) The distribution percentage of sample in each subtype.

After using z-score scaling to ensure consistent scaling across variables, a two-cluster solution was selected based on the highest silhouette coefficient, indicating optimal cluster separation (Fig. 1A). Furthermore, dimension reduction was executed using PCA, revealing three principal components that accounted for 73 % of the variations in the data (Fig. 1B). The visualization of K means clustering was depicted in a three-dimensional space (Fig. 1C). Cluster analysis identified two subtypes, with Subtype 1 making up 48.3 % of the sample (Fig. 1D).

### The baseline characteristics of ID subtypes

**Fig. 2:**
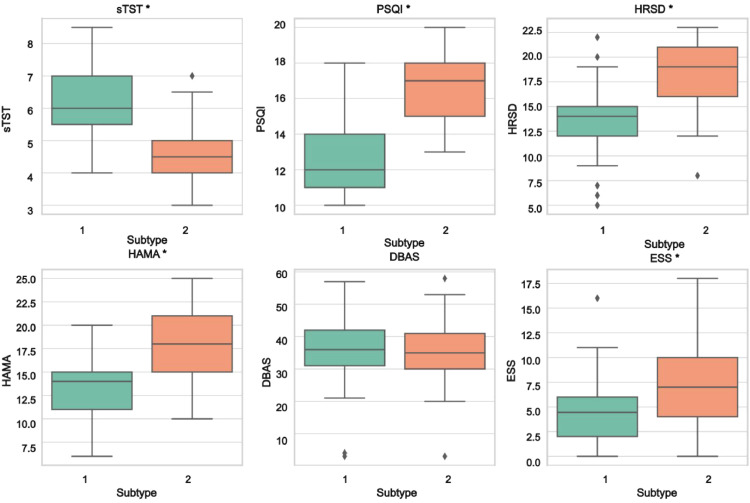
The baseline sleep and emotional characteristics of the data-driven ID subtypes. * indicates a statistically significant difference between Subtype 1 and Subtype 2 (*p* < 0.05).

Compared to Subtype 1, Subtype 2 demonstrated significantly shorter self-reported total sleep time (sTST: 4.67 vs. 6.09 h, *t* = 8.31, *p* < 0.01), worse sleep quality (PSQI: 16.59 vs 12.74, *t*=−9.90, *p* < 0.01), more severe depressive symptoms (HRSD: 18.47 vs 13.21, *t*=−8.37, *p* < 0.01), anxiety symptoms (HAMA: 17.47 vs 13.46, *t*=−6.23, *p* < 0.01), and sleepiness (ESS: 6.98 vs 4.51, *t*=−3.54, *p* < 0.01) (Fig. 2). No significant differences were found between Subtype 1 and Subtype 2 in BMI (*T* = 0.03, *p* = 0.976), age (35.18 ± 9.96 vs. 34.92 ± 10.13; *T* = 0.13, *p* = 0.900), sex (63.1 % vs. 62.2 % female; *U* = 1719.5, *p* = 0.903), education level (16.46 ± 2.28 vs. 16.89 ± 2.25 years; *U* = 1899.5, *p* = 0.100), marital status (45.21 % vs. 45.95 % married; *U* = 1822.0, *p* = 0.611), employment status (χ² = 1.35, *p* = 0.2445), hypnotic use (*U* = 1524.0, *p* = 0.179), or dysfunctional beliefs about sleep (DBAS: 34.95 vs. 34.79; *T*=−0.08, *p* = 0.936).

### The impact of subtypes on the efficacy of CBT-I

**Fig. 3:**
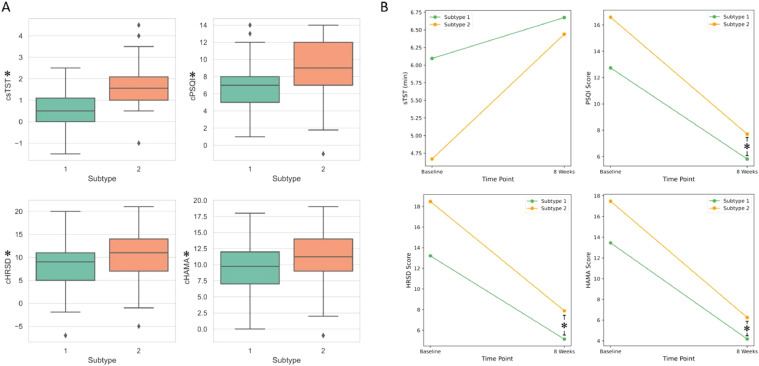
Characteristics and changes in sleep and emotion between ID Subtypes Following CBT-I Treatment (A) The changes in sleep and emotional characteristics of the data-driven ID subtypes after CBT-I. (B) Sleep and emotional characteristics of ID subtypes at baseline and 8 weeks post-CBT-I. * indicates a statistically significant difference between Subtype 1 and Subtype 2 (*p* < 0.05).

After 8 weeks of CBT-I or CBT-I plus intervention, both subtypes displayed changes in sleep and emotional characteristics (Fig. 3A). Here, the prefix “c” indicates “change” and represents the difference between baseline and post-treatment values for each variable. Participants in Subtype 2 had a larger increase in sleep duration (csTST in Subtype 2 vs Subtype 1: 1.77 vs 0.58 h, *t* = −7.18, *p* < 0.01) and greater improvement in self-reported sleep quality (cPSQI score in Subtype 2 vs.1: 8.88 vs. 6.92, *t* = −3.57, *p* < 0.001). Participants in Subtype 2 demonstrated significant larger reductions in depressive symptoms (cHRSD in Subtype 2 vs. 1: 10.59 vs. 8.07, *t* = −2.71, *p* = 0.008) and anxiety symptoms (cHAMA in Subtype 2 vs. 1: 11.22 vs. 9.28, *t* = −2.56, *p* = 0.012). No significant difference in self-reported sleep duration was observed between participants in Subtype 1 and Subtype 2 at 8 weeks (6.68 h vs 6.44 h, *p* = 0.119). However, participants in Subtype 1 exhibited significantly better outcomes in terms of sleep quality (PSQI_8w: 5.81 vs 7.71, *p* < 0.01), depression levels (HRSD_8w: 5.14 vs 7.89, *p* < 0.01), and anxiety levels (HAMA_8w: 4.18 vs 6.25, *p* < 0.01) compared to those in Subtype 2. These results, as visualized in Fig. 3B, underscore the more pronounced treatment benefits experienced by Subtype 2 participants, particularly in terms of sleep and emotional improvements.

### Efficacy differences of CBT-I and CBT-I plus between subtypes

In our analysis, we found no significant differences in baseline characteristics between the groups receiving either CBT-I alone or CBT-I plus within both Subtype 1 and Subtype 2. This suggests homogeneity in initial sleep and emotional conditions across the two treatment modalities. Detailed statistical results, including t-values and p-values for measures such as sTST, PSQI, HRSD, and HAMA, are provided in Supplemental Table 1.

**Fig. 4:**
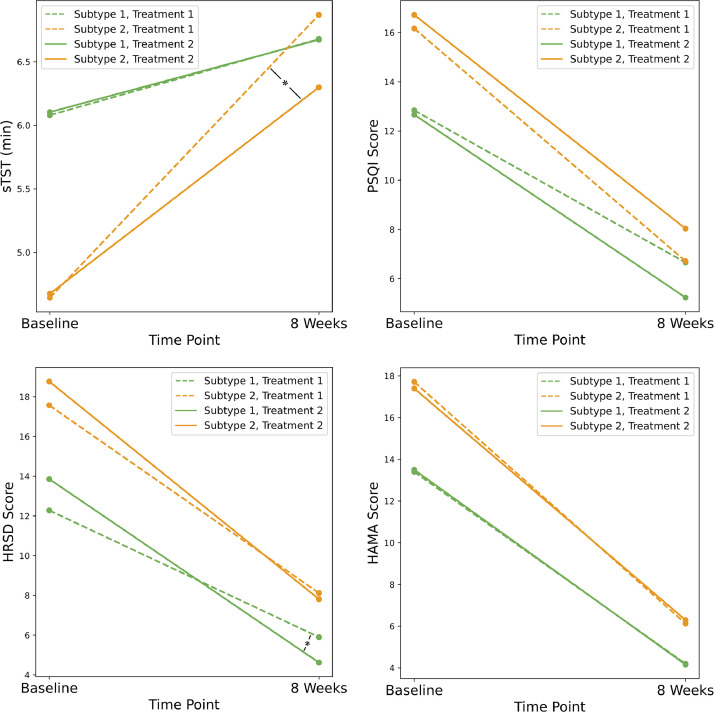
Differential Impact of CBT-I vs. CBT-I plus on Sleep and Emotion Outcomes Across Subtypes. * indicates a statistically significant difference between Subtype 1 and Subtype 2 from baseline to 8 weeks (*p* < 0.05).

In the results presented in Fig. 4, within Subtype 1, comparisons between CBT-I and CBT-I plus revealed no significant differences in csTST (*t* = 0.139, *p* = 0.890), cPSQI (*t* = −1.94, *p* = 0.057), or cHAMA (*t* = −0.136, *p* = 0.893). However, a statistically significant difference was observed in the reduction of depressive symptoms (cHRSD: *t* = −2.48, *p* = 0.016), indicating that CBT-I plus was more effective in improving depressive symptoms among participants in Subtype 1. For Subtype 2, the analysis indicated that CBT-I resulted in a greater increase in csTST than CBT-I plus (*t* = 2.01, *p* = 0.049), but no significant differences were found in cPSQI (*t* = 0.74, *p* = 0.462), cHRSD (*t* = −0.892, *p* = 0.376), or cHAMA (*t* = 0.364, *p* = 0.717) between the two interventions. The findings indicated that CBT-I plus more effectively reduced depressive symptoms in Subtype 1, while CBT-I enhanced self-reported sleep duration more in Subtype 2, with no significant differences in other measures between treatments.

## Discussion

The heterogeneity of ID suggests that classifications relying on a single feature inadequately capture the complexity of the condition (Benjamins et al., 2017; Crawford et al., 2017). Our study utilizes data on multiple symptoms of insomnia disorder (ID) subtyping, identifying two unique symptom-based patterns of ID. These findings reveal differing trajectories in these subtypes following cognitive-behavioral therapy for insomnia (CBT-I) and its augmented versions (CBT-I plus), providing valuable insights for personalized treatment strategies.

Compared to other data-driven insomnia disorder (ID) subtyping studies, our research incorporates sleep and emotional characteristics commonly assessed in clinical settings. We identified two main ID subtypes based on sleep and emotional features: Subtype 1 is characterized by longer self-reported sleep duration, better sleep quality, and lower levels of depression, anxiety, and sleepiness, while Subtype 2 is characterized by shorter self-reported sleep duration, poorer sleep quality, and higher levels of depression, anxiety, and sleepiness. Various studies have revealed unique patterns of ID severity through data-driven approaches. For instance, Crawford et al. identified two severe subtypes and one milder insomnia subtype with distinct sleep quality and sleepiness characteristics using Latent Profile Analysis (Crawford et al., 2017). Sforza et al. categorized insomnia patients by depression, anxiety and stress symptoms into subtypes and found that depression and stress symptoms ought to be deemed as risk factors of long-term CBT-I therapeutic efficacy (Sforza et al., 2021). Laar et al. have also identified subtypes with varying self-reported TST and depression levels, where the "moderate insomnia with low psychopathology" and "severe insomnia with moderate psychopathology" subtypes closely resemble the findings of our study (Van De Laar et al., 2017). However, our research has not further subtyped the more severe Subtype 2, possibly due to the lack of physiological TST and personality and life history characteristics. The subtype with shorter sleep duration exhibits more severe biological features of insomnia; However, it remains unclear whether short sleep duration improves or worsens CBT-I outcomes. Although Blanken et al. found differences in reward sensitivity and sleep reactivity among insomnia subtypes with varying distress levels, evidence supporting these the use of these additional differences to guide treatment decisions is still lacking (Blanken et al., 2019). Hence, our study elucidates the severity of ID based on sleep and emotional feature-based subtyping.

This study found that incorporating emotion-targeted treatment modules into CBT-I is more effective for a subtype of ID with milder severity. Co-occurring depression and anxiety with insomnia are common in clinical settings (Morin et al., 2023), and evidence suggests that CBT-I can alleviate both ID and its comorbid depressive and anxiety symptoms (Blom et al., 2017; Ho et al., 2020; Belleville et al., 2011). However, depression and anxiety as residual symptoms warrant attention due to their potential indication of future depressive episodes (Duffy et al., 2023). A study conducted by Zhang and colleagues has demonstrated that baseline depressive and anxiety symptoms can diminish the treatment's effectiveness of CBT-I, suggesting the importance of addressing these comorbid conditions alongside CBT-I (Zhang et al., 2023). Incorporating classic CBT emotional processing techniques into CBT-I plus, aimed at challenging depression-specific negative thoughts, provides additional therapeutic benefits for Subtype 1.

However, our study demonstrated that Subtype 2 showed greater improvements in sleep quality and emotional states in response to CBT-I compared to CBT-I plus, despite the potential ceiling/floor effect from the higher severity of depression and anxiety in this subtype. According to the distribution of baseline scores and post-treatment changes, Subtype 2′s improvements were not exclusively concentrated at the scale extremes, implying that the observed differences likely represent genuine therapeutic benefits rather than measurement artifacts. This aligns with previous research suggesting that more severe insomnia subtypes may not respond better to treatments that involve additional therapeutic components. Sadler et al. conducted an RCT using a treatment similar to the CBT-I plus approach in our study for comorbid insomnia and depression (Sadler et al., 2018). They found that the efficacy was comparable to standard CBT-I, possibly due to the added level of complexity in CBT-I plus, which may have limited its benefits for subtypes with more severe depression. Additionally, Subtype 2 patients may have more profound sleep dysregulation, so that they may require more targeted biological interventions. Circadian rhythm disruption, a key etiology in mood and sleep disorders, might complicate the treatment efficacy for subtype 2 (Crouse et al., 2021). Interventions targeting circadian rhythms could benefit depressive symptoms associated with ID (Dekker et al., 2020). A study by Leerssen et al. on adding Circadian Rhythm Support intervention to CBT-I indicated the prevention of worsening depressive symptoms in ID (Leerssen et al., 2022). Similarly, a protocol by Schmid et al. suggested that Light therapy as an add-on to CBT-I could aid in alleviating depression and sleepiness (Schmid et al., 2022). Implementing an insomnia stepped care model, which tailors treatment intensity and adjunctive interventions according to clinical severity and comorbidities, may further optimize these approaches (Baglioni et al., 2023). Therefore, selecting different add-on therapies for depressive symptoms presented by different ID subtypes represents a promising personalized treatment model.

This study has several limitations. First, the modest sample size restricted the number of variables used for clustering. Future studies with larger samples could incorporate additional sleep indices and validate the clustering. Second, the absence of polysomnographic measures precluded verification of physiological changes influencing insomnia. Finally, while this single clinical trial was conducted in a specific population and included only adults aged 18–60, subtypes of insomnia have been explored in elderly (Yu et al., 2017) and pediatric (Bruni et al., 2018) populations. Replication in larger and more diverse cohorts is therefore needed to confirm the generalizability of these findings. Despite these limitations, this study provides valuable insights into the heterogeneity of insomnia disorder and underscores the importance of personalized treatment approaches based on data-driven subtypes.

## Conclusions

In conclusion, our study delineates a data-driven classification of ID subtypes with different sleep and emotional profiles. The symptom-specific superiority of CBT-I plus advocates for the potential of emotion-targeted interventions in addition to CBT-I. These findings underscore the value of personalized interventions in optimizing insomnia disorder treatment and highlight their potential integration into stepped care models.

## Author contributions

Dongbin Lyu and Ruiyi Qian contributed equally to the study design. Dongbin Lyu, Ruiyi Qian, Fangmei Ge, Yang Wang, Hongyan Wang, Yating Zhao, Hui Han, Ruyun Liu, Yutong Liu, Yiling Chen, Caojun Ji, Xin Luo, and Tianhong Zhang were responsible for data collection, processing, and curation. Dongbin Lyu, Ruiyi Qian, and Yang Wang completed the data analysis. Dongbin Lyu drafted the manuscript. Yue Leng and Zeping Xiao provided critical insights and pivotal support in the manuscript's composition, data interpretation, and preparatory stages. Jie Zhang, Chengmei Yuan, Zeping Xiao, and Tianhong Zhang conceptualized and supervised the study's data collection, logistics, and analysis. All authors reviewed and approved the final manuscript.

## Declaration of competing interest

The authors declare that they have no known competing financial interests or personal relationships that could have appeared to influence the work reported in this paper.

## Appendix Supplementary materials

Image, application 1

Image, application 2
