# Supplementary material for: Exploring the associations between data-driven insomnia disorder combined with mild anxiety or/and depressive symptoms and the efficacy of Cognitive-Behavioral Therapy for insomnia
Source: Int J Clin Health Psychol. 2025 Apr 8;25(1):100562. doi: 10.1016/j.ijchp.2025.100562 (PMC12005858; doi:10.1016/j.ijchp.2025.100562)
Supplement: Supplementary file 1 [file mmc1.docx]

**Components of CBT-I and CBT-I plus**

**Cognitive Behavioral Therapy for Insomnia (CBT-I)**

Standard CBT-I is composed of five main modules:

1. **Stimulus Control Technique**

Based on classical and operant conditioning principles, this technique aims to extinguish negative associations between the bed and wakefulness while reinforcing a stable **sleep–wake schedule**. Patients are instructed to go to bed only when they feel sufficiently sleepy or at a prescribed bedtime.

2. **Sleep Restriction**

By restricting or limiting the patient’s time in bed to more closely match their recorded **total sleep time**, this method helps realign the individual’s **sleep physiology and circadian rhythm**. As the total sleep time extends, the patient gradually readjusts the amount of time spent in bed.

3. **Sleep Hygiene Education**

Patients learn how factors such as environmental conditions (lighting, noise), dietary elements (nicotine, alcohol, caffeine), and exercise can affect their sleep. Through this psychoeducation, patients identify which behaviors may be interfering with sleep.

4. **Relaxation Training**

Used to reduce intrusive thoughts and **physiological hyperarousal**, which can disrupt sleep.

5. **Cognitive Restructuring for Sleep**

Patients are guided to identify and examine **negative automatic thoughts** and core beliefs about sleep. Through discussion, challenge, and behavioral experiments, patients learn to modify these **maladaptive cognitions**.

**Table 1. CBT-I Protocol**

| Session | Content |
| --- | --- |
| 1 | Establish therapeutic rapport; introduce CBT-I; provide sleep hygiene education; instill hope and motivation; teach relaxation exercises. |
| 2 | Review sleep mechanisms; analyze causes of insomnia; introduce sleep restriction; develop a sleep schedule; establish commitment; practice relaxation. |
| 3 | Evaluate treatment benefits and adherence; address barriers to completing sleep plans; further adjust sleep schedule; promote behavioral change; practice relaxation. |
| 4 | Evaluate treatment benefits and adherence; adjust sleep schedule; introduce stimulus control technique; conduct mid-term summary; practice relaxation. |
| 5 | Continue adjusting sleep plan; discuss factors hindering adherence; ensure compliance with the schedule; practice relaxation. |
| 6 | Refine the sleep plan; introduce cognitive restructuring for dysfunctional sleep beliefs; practice relaxation. |
| 7 | Continue adjusting the sleep plan; further cognitive restructuring; prepare for treatment termination; practice relaxation. |
| 8 | Assess treatment outcomes; discuss medication-related concerns; address relapse prevention; conclude therapy. |

**Enhanced Cognitive Behavioral Therapy for Insomnia (CBT-I plus)**

CBT-I plus augments standard CBT-I by incorporating targeted interventions for patients with coexisting anxiety and depressive symptoms. In addition to preserving the five core modules of standard CBT-I, CBT-I plus includes the following enhancements:

**1. Education on the Relationship Between Insomnia and Anxiety/Depression**

Patients learn about the fundamental aspects of anxiety and depressive states, and how these conditions interact with insomnia.

**2. Expanded Cognitive Restructuring**

Participants undergo added assessments for anxiety and depression—such as identifying anxiety symptoms and keeping an “anxiety monitoring diary”—while learning strategies to recognize and correct negative automatic thoughts specifically related to anxiety and depression.

**3. Additional Cognitive Reframing Techniques**

Patients learn new methods to dispute unhelpful cognitions, develop alternative coping statements, and create personalized mantras or counter-thoughts.

**Table 2. Enhanced CBT-I (CBT-I plus) Protocol**

| Session | Content |
| --- | --- |
| 1 | Establish therapeutic rapport; introduce the structured, enhanced psychotherapy tool (CBT-I plus) for insomnia with comorbid depression/anxiety; provide sleep hygiene education; instill hope and motivation; teach relaxation exercises. |
| 2 | Explain sleep mechanisms; analyze insomnia etiology; teach sleep restriction; create a sleep plan; establish compliance; practice relaxation. |
| 3 | Evaluate treatment benefits and adherence; address obstacles to plan execution; adjust the sleep plan; reinforce behavior change; practice relaxation. |
| 4 | Assess treatment benefits and adherence; refine the sleep plan; teach stimulus control technique; summarize progress to date; practice relaxation. |
| 5 | Continue refining the sleep plan; identify dysfunctional beliefs about sleep and recognize anxiety symptoms; reframe negative automatic thoughts related to anxiety. |
| 6 | Modify the sleep plan; challenge dysfunctional sleep beliefs; identify depressive symptoms and reframe negative automatic thoughts related to depression. |
| 7 | Continue adjusting the sleep plan; further cognitive restructuring; prepare for treatment termination. |
| 8 | Evaluate treatment outcomes; discuss relapse prevention; conclude therapy. |
